# Endometrial carcinosarcoma with multi-classifier molecular signature (MMRd-p53abn): A case report

**DOI:** 10.18632/oncoscience.665

**Published:** 2026-07-29

**Authors:** Aparna Jarathi, Akhila Pagolu, Naina Kumar, Shrinivas Somalwar, Sai Swetha Banka, Nireesha Bukke, Chandramouli Ramalingam

**Affiliations:** ^1^Department of Obstetrics and Gynaecology, All India Institute of Medical Sciences, Bibinagar, Hyderabad, Telangana 508126, India; ^2^Department of Pathology and Laboratory Medicine, All India Institute of Medical Sciences, Bibinagar, Hyderabad, Telangana 508126, India; ^3^Department of Radiation Oncology, All India Institute of Medical Sciences, Bibinagar, Hyderabad, Telangana 508126, India

**Keywords:** chemotherapy, pole mutant, MMRd-P53abn, postmenopausal, uterine carcinosarcoma

## Abstract

The uterine carcinosarcoma is a rare biphasic Malignant Mixed Mullerian Tumour (MMMT). It is a highly aggressive endometrial cancer phenotype, consisting of both epithelial and mesenchymal components. The presented case refers to a 65-year-old, P6L3 postmenopausal woman with postmenopausal bleeding and white discharge per vagina for one month with vulval leukoplakia. The ultrasound showed a thickened endometrium measuring 15 mm, and the Magnetic Resonance Imaging showed the uterus measuring 7.4 × 4.5 × 6 cm. The endometrium appeared heterogeneous, with a hyperintense lesion measuring 4.8 × 2.3 cm on T2W1. The myometrium appeared normal, and neither of the ovaries was visualized. The patient underwent surgery. The histopathological and immunohistochemical analysis suggested that high-grade Endometrial Carcinosarcoma with dual classifier positivity (MMRd-p53abn) more closely resembled single-classifier MMRd (Mismatch repair deficiency) than p53abn, with a good prognosis. Post-surgery, the patient was followed up on every three months. The patient received 6 cycles of adjuvant chemotherapy. Considering the highly invasive nature of uterine carcinosarcomas, the timely detection of this cancer by imaging, pathology findings and molecular classification is of extreme importance to improve the patient’s survival and for management outcome.

## INTRODUCTION

Uterine Carcinosarcoma (also known as malignant mixed Mullerian tumor, MMMT) is a rare malignancy accounting for less than 5% of all uterine cancers. Despite its rarity, it is responsible for 15% of deaths from uterine malignancies. Carcinosarcomas are staged the same as Endometrial carcinomas [1]. They exhibit aggressive behaviour and have a poor prognosis, even in the early stages. 5-year survival rate of stage I: 55%, stage II: 37%, stage III: 25%, stage IV: 10% [2].

In recent years, immunohistochemistry (IHC) and molecular classification have become essential for tailoring targeted therapies for patients and differentiating endometrial carcinoma subtypes. IHC enables the detection of abnormal p53 expression and mismatch repair deficiency (MLH-1, MSH-2, MSH-6, and PMS-2), while PCR (polymerase chain reaction) and sequencing methods are used to identify pathogenic POLE (DNA Polymerase Epsilon) mutations and to assess the presence of microsatellite instability (MSI) [2]. Importantly, tumors with mismatch repair deficiency (MMRd) and MSI-high status have demonstrated increased responsiveness to immune checkpoint inhibitors, highlighting the emerging role of immunotherapy in the management of selected endometrial carcinomas and related high-grade malignancies.

Application of molecular classification in all cases of endometrial carcinoma is encouraged to support prognostic risk-group stratification and to inform decisions on adjuvant or systemic treatment. POLEmut tumors have a favourable prognosis. MMRd and NSMP indicate an intermediate prognosis and therefore do not alter the stage, while p53abn indicates a poor prognosis and is typically associated with high-grade serous histology [3]. Multiple molecular classifiers are defined by the presence of more than one molecular feature, with an incidence of 1.8 to 14.3%. The most common is MMRd-p53abn, followed by POLEmut- MMRd, POLEmut-p53abn, and POLEmut-MMRd-p53abn. For genomic features and survival outcomes, when multiple molecular classifiers are present, tumors with POLEmut or MMRd and a secondary p53 abnormality should be staged according to the POLEmut or MMRd category [4]. In patients with early-stage endometrial cancer (stages IA, IB and II), molecular classification plays a crucial role in risk stratification, enabling downstaging (e.g., stage IAm for POLEmut tumors) or upstaging (e.g., stage IICm for p53abn tumors).

## CASE REPORT

**Figure 1:**
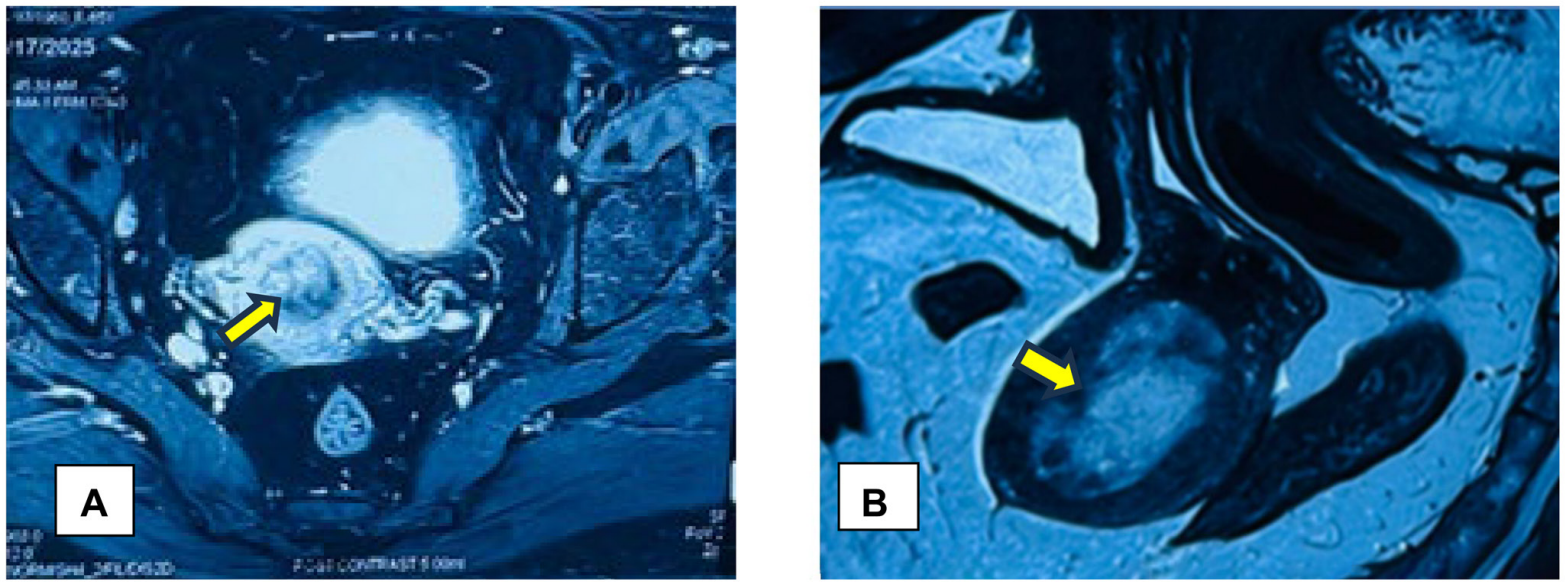
(**A**) MRI showing diffusion restriction of the lesion in the endometrium (Yellow Arrow). (**B**) heterogeneous mass inside the uterus on sagittal T2W (Yellow Arrow).

**Figure 2:**
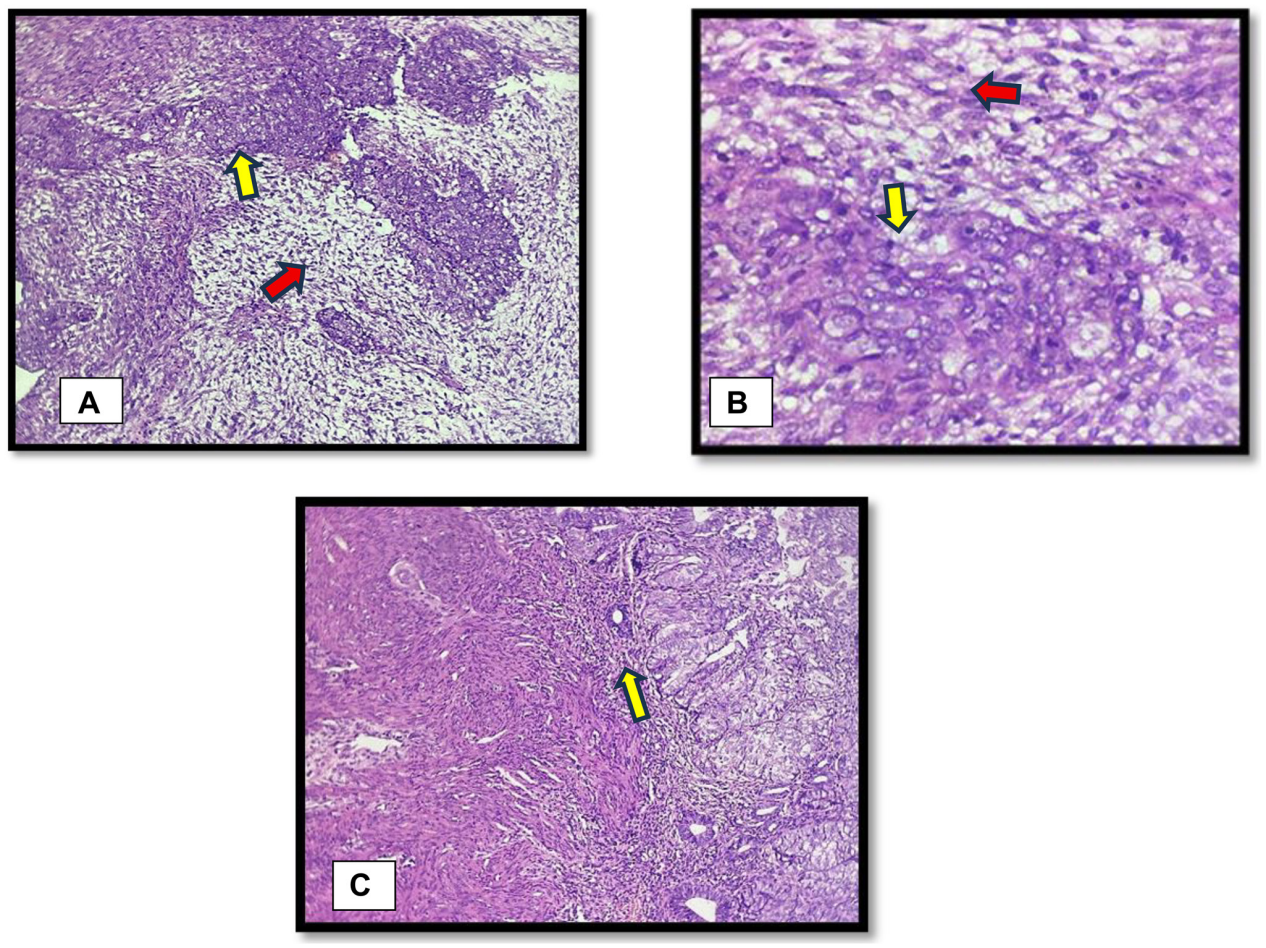
(**A**) (10X) and (**B**) (40X) HPE: Biphasic pattern showing Epithelial component (yellow arrow); sarcomatous component (red arrow). (**C**) The tumour is restricted to the endometrium (yellow arrow).

A 64-year-old postmenopausal woman (P6L3), menopausal for 20 years, presented to the gynaecology outpatient department with complaints of intermittent postmenopausal bleeding and non-foul-smelling white discharge per vagina associated with itching for the past month. The patient acknowledged weight loss over the past 6 months (BMI 27.2 kg/m²) and reported normal bowel and bladder habits. The general physical examination was unremarkable. In the pelvic ultrasonography, the endometrial thickness was 15 mm, and MRI of pelvis (Figure 1A, 2B) showed a normal-sized uterus (7.4 × 4.5 × 6 cm) with T2 heterogeneously hyperintense signal intensity lesion measuring 4.8 × 2.3 cm in the endometrium with a patchy diffusion restriction with an ADC value of 0.9 × 10^−3^ mm^2^/s with progressively heterogeneous enhancement that is less than that of the surrounding myometrium suggestive of endometrial malignancy. The junctional zone and myometrium showed a normal signal pattern. The Pap smear was negative for intraepithelial malignancy. A vulval biopsy done for vulval leukoplakia showed non-specific findings with a mild increase in collagen. Fractional curettage and endometrial biopsies revealed sections showing abundant tumour tissue having a biphasic pattern with malignant endometroid-type glands and an abundant malignant-appearing stromal component. In (Figure 2A–2C), both epithelial and stromal elements exhibited pleomorphism and increased mitosis with large areas of necrosis and haemorrhage, features suggestive of carcinosarcoma.

**Figure 3:**
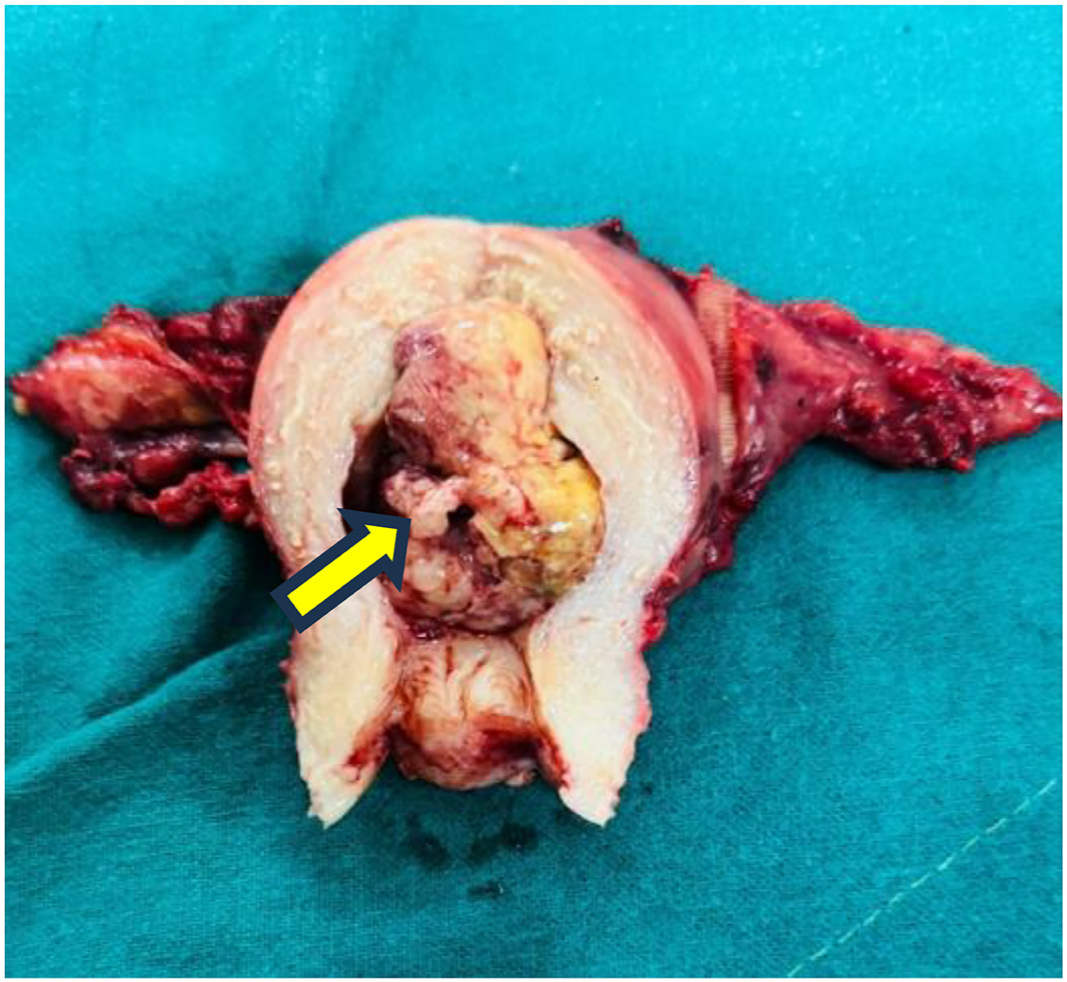
Polypoidal growth filling the uterine cavity (Yellow arrow).

The patient underwent a total abdominal hysterectomy with bilateral salpingo-oophorectomy with bilateral pelvic lymph node dissection, omentectomy, peritoneal biopsy, and peritoneal fluid cytology was sent. Intraoperatively, the uterus was uniformly enlarged to the size of an 8-week. The right lower segment of the uterus near the isthmus and cervicovaginal junction was friable and highly vascular. Bilateral tubes, ovaries, liver, spleen, intestine, and peritoneum were grossly normal-looking. Gross examination of the uterus showed an irregular, fleshy, polypoidal growth of 5 × 2.5 × 2 cm, attached a bit to the endometrium (Figure 3). It appeared grossly confined to the endometrium with no myometrial invasion. The cut surface of the growth shows grey-white to haemorrhagic, necrotic, and glistening areas.

Based on histopathology, it was a high-grade endometroid homologous uterine carcinosarcoma. Peritoneal fluid cytology was negative for malignancy. Bilateral tubes, ovaries, cervix, peritoneum, and omentum were negative for tumour, with no lymphovascular space invasion (LVSI) or lymph node involvement.

The American Joint Committee on Cancer (AJCC, eighth edition) stage was pT1a, pN not applicable. According to the International Federation of Gynecology and Obstetrics (FIGO 2023), it was staged as FIGO stage IC [3]. The postoperative period was uneventful. Molecular classification was performed using next-generation sequencing (NGS), which revealed a TP53 abnormality and an MMRd (MSH6) frameshift mutation.

Follow-up: Post-surgery, the patient was followed up on every 3 months. Patient received 6 cycles of adjuvant chemotherapy (carboplatin/paclitaxel). The patient had one episode of Transient Ischemic Attack after 6 cycles of chemotherapy and was managed medically and recovered with no residual disease. She was scheduled for a Positron Emission Tomography scan.

## DISCUSSION

Uterine carcinosarcoma (UCS) is a rare and aggressive high-grade endometrial carcinoma [5]. Approximately 30–40% of cases show extrauterine spread, and over 10% have distant metastasis, most frequently involving lungs and lymph nodes [1]. It is more common in the black ethnicity [6]. Other risk factors include obesity, nulliparity, a history of pelvic radiation, and prior tamoxifen use [3]. The median age ranges from 62 to 67 years [7].

The most common clinical manifestations of UCS are an enlarged intrauterine mass, abnormal uterine bleeding, and pelvic pain [8]. Ultrasound is the first-line diagnostic modality, and UCS appears as a hyperechoic mass relative to the surrounding endometrium. MRI plays an essential role as the primary imaging and staging modality, with 80% accuracy. UCS appears as a mass exhibiting low-to-isointense signal on T1-weighted images, with areas of high signal, which is considered highly specific, and high- or mixed signal on T2-weighted images, with similar correlation findings in our case. Mild to moderate contrast enhancement is a key distinguishing feature of carcinosarcoma compared to other malignant uterine tumors. Computed tomography (CT) can be helpful for preoperative evaluation of the extent of extrauterine spread [9]. Gloria Huang et al. reported that preoperative elevation of CA-125 is a marker of extrauterine disease and deep myometrial invasion in patients with UCS [10].

The histological diagnosis of carcinosarcoma requires the presence of a distinct biphasic tumor (Epithelial and Stromal). Four principal hypotheses have been proposed regarding its cellular origin. The conversion/divergence hypothesis is most common, suggesting that the sarcomatous component results from a metaplastic sarcomatous transformation of the epithelial component. The stromal (mesenchymal) component may be homologous (leiomyosarcoma, stromal sarcoma, fibrosarcoma) or heterologous (chondrosarcoma, rhabdomyosarcoma, osteosarcoma, liposarcoma). Heterologous mesenchymal components are often associated with a poorer prognosis than those with homologous elements [1, 2]. In our patient, only a homologous stromal component was present.

**Figure 4:**
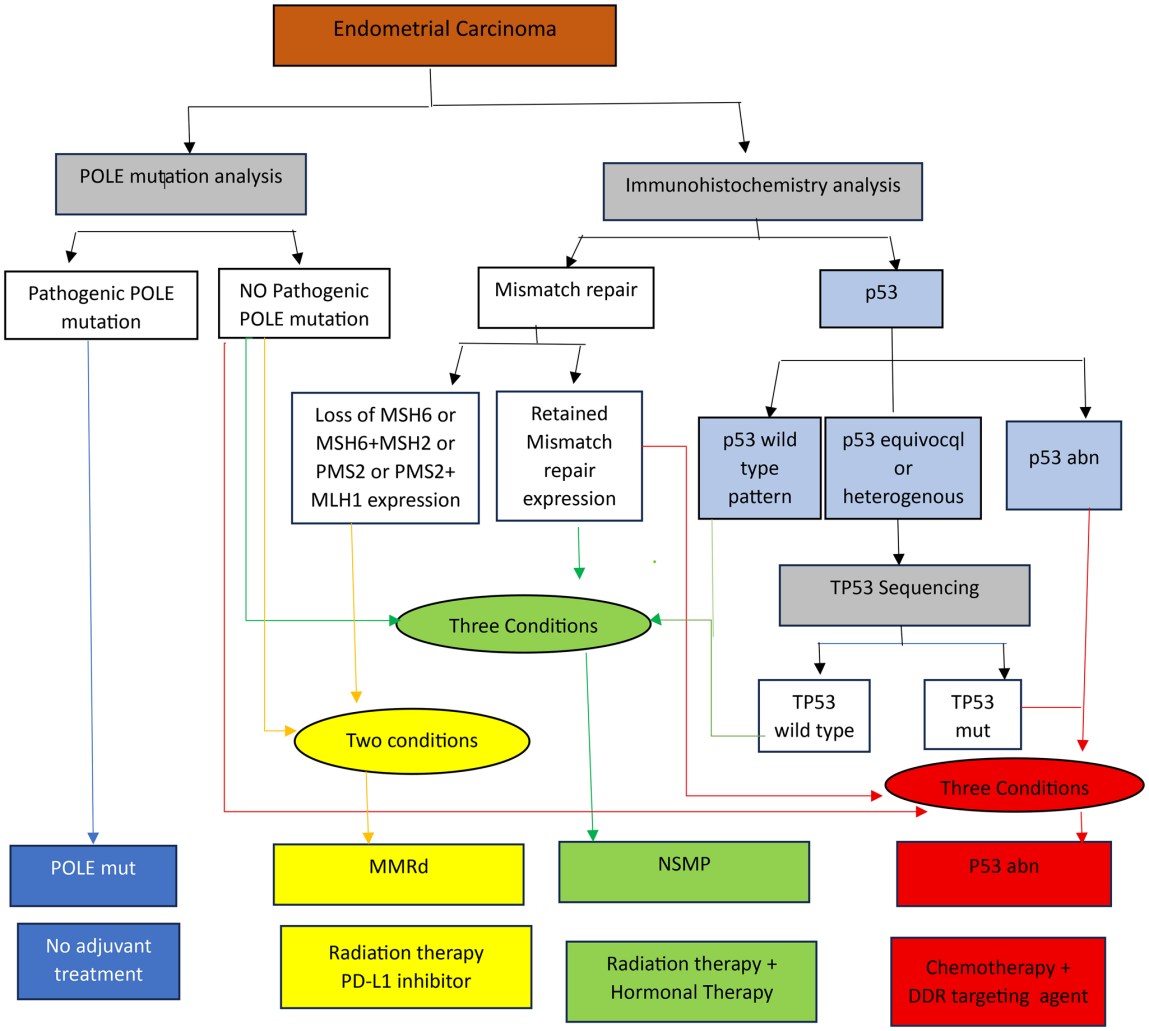
Algorithm for assessment of molecular classification of endometrial carcinoma. Abbreviations: POLEmut: polymerase epsilon-ultramutated; MMRd; mismatch repair deficient; NSMP: no specific Endometrial carcinoma; PD-L1: programmed death ligand-1; DDR: DNA damage response.

The Cancer Genome Atlas (TCGA) classified endometrial carcinoma into four groups: POLEmut (10.9%), MMRd (2.4% to 26.1%), p53abn (50% to 91%), and no specific molecular profile (NSMP) (3% to 19%). POLE-ultramutated tumors are identified by sequencing of the POLE exonuclease domain, microsatellite instability–high (MSI-H)/hypermutated tumors are detected by mismatch repair protein immunohistochemistry (MMRd), and copy-number high tumors are identified by aberrant p53 immunohistochemistry (p53abn). Endometrial carcinomas lacking these molecular features are classified as no specific molecular profile (NSMP) and broadly correspond to the copy-number–low group. An algorithm for the molecular classification of endometrial carcinoma is illustrated in Figure 4 [11].

In early endometrial cancer, the presence of POLE mutations or p53abn now modifies FIGO staging. A POLE-mutated endometrial carcinoma limited to the uterine corpus, or extending to the cervix, is classified as Stage I AmPOLEmut, irrespective of lymphovascular space invasion (LVSI) or histological subtype. In contrast, a p53-abnormal endometrial carcinoma confined to the uterus, with any degree of myometrial invasion and with or without cervical involvement, is classified as Stage II Cmp53abn, regardless of LVSI. In advanced endometrial cancer, staging remains unchanged even after molecular classification. Stage III and IV tumors with p53 abnormalities should be recorded as Stage III mp53abn and Stage IV mp53abn, respectively. Similarly, Stage III and IV tumors with MMR deficiency should be documented as Stage III mMMRd and Stage IV mMMRd, respectively [3].

According to the European Society of Gynaecological Oncology/European Society for Radiotherapy and Oncology/European Society of Pathology (ESGO/ESTRO/ESP) guidelines, p53-abnormal and NSMP tumors are associated with a worse prognosis, whereas the prognostic significance of MSI-H/MMRd tumors remains less clearly defined [11, 12]. MMRd and NSMP carcinomas constitute the majority of endometrioid endometrial cancers with an intermediate prognosis, between POLE-mutated tumors (excellent prognosis) and p53-abnormal carcinomas [11].

León-Castillo et al. analysed 137 tumors with multiple molecular classifiers and identified 64 cases with dual MMRd-p53abn positivity. These were predominantly early-stage, high-grade endometrioid carcinomas, usually without lymphovascular space invasion, and commonly showed loss of MSH6 with or without MSH2, or isolated PMS2 loss [4]. In the present case, POLE, MLH1, MSH2, and PMS2 were negative, while TP53 mutation and an MSH6 frameshift mutation were identified, categorising the tumor as a dual-classifier subtype (MMRd-p53abn). MMRd-p53abn more closely resembled a single-classifier MMRd. Sub clonal p53 expression was more frequent in multiple classifiers than in p53abn single classifiers. The current adjuvant treatment guidelines are based on the prognostic risk group (Table 1) [11].

**Table 1:** Definition of prognostic risk groups [11]

Risk group	Molecular classification unknown	Molecular classification known
**Low**	Stage IA endometrioid + low-grade^‡^ + LVSI negative or focal	Stage I–II POLEmut endometrial carcinoma, no residual disease Stage IA MMRd/NSMP endometrioid carcinoma + low-grade^‡^ + LVSI negative or focal
**Intermediate**	Stage IB endometrioid + low-grade^‡^ + LVSI negative or focal Stage IA endometrioid + high-grade^‡^ + LVSI negative or focal Stage IA non-endometrioid	Stage IB MMRd/NSMP endometrioid carcinoma + low-grade^‡^ + LVSI negative or focal Stage IA MMRd/NSMP endometrioid carcinoma + high-grade^‡^ + LVSI negative or focal Stage IA p53abn and/or non-endometrioid
**High–intermediate**	Stage I endometrioid + substantial LVSI regardless of grade and depth of invasion Stage IB endometrioid high-grade^‡^ regardless of LVSI status Stage II	Stage I MMRd/NSMP endometrioid carcinoma + substantial LVSI regardless of grade and depth of invasion Stage IB MMRd/NSMP endometrioid carcinoma high-grade^‡^ regardless of LVSI status Stage II MMRd/NSMP endometrioid carcinoma
**High**	Stage III–IVA with no residual disease Stage I–IVA non-endometrioid	Stage III–IVA MMRd/NSMP endometrioid carcinoma with no residual disease Stage I–IVA p53abn endometrial carcinoma with myometrial invasion, with no residual disease Stage I–IVA NSMP/MMRd serous, undifferentiated carcinoma, carcinosarcoma with myometrial invasion, with no residual disease
**Advanced metastatic**	Stage III–IVA with residual disease Stage IVB	Stage III–IVA with residual disease of any molecular type Stage IVB of any molecular type

Abbreviations: LVSI: lymphovascular space invasion; MMRd: mismatch repair deficient; NSMP: non-specific molecular profile; p53abn: p53 abnormal; POLEmut: polymerase-mutated. ^‡^According to the binary FIGO grading, grade 1 and grade 2 carcinomas are considered as low-grade and grade 3 carcinomas are considered as high-grade.

Surgical excision is the treatment of choice for UCS -Total hysterectomy, bilateral salpingo-oophorectomy, and pelvic and para-aortic lymphadenectomy [7]. Carcinosarcomas should be managed as high-risk carcinomas, with either concurrent external beam radiotherapy (EBRT) and adjuvant chemotherapy or sequential chemotherapy and radiotherapy. Chemotherapy alone may be considered as an alternative option [11].

However, based on the results from the GOG-232 B and GOG-261 trials and the National Comprehensive Cancer Network (NCCN) guidelines, adjuvant therapy with carboplatin/paclitaxel is now recommended as the preferred first-line treatment for endometrial carcinosarcoma. Ifosfamide/paclitaxel and cisplatin/paclitaxel regimens remain alternative options (for instance, in cases of hypersensitivity to carboplatin) [13]. Tumours with dMMR are sensitive to immune checkpoint inhibitors, making immunotherapy an effective treatment option that can improve outcomes [14].

Clinical follow-up includes physical examination, vaginal cytology, recommended every 3 months for the first 2 years, then every 6 months for 5 years. If there is clinical suspicion of recurrence or metastasis, imaging with abdominal-pelvic or chest CT is advised. For patients with suspected or confirmed distant metastases, whole-body PET-CT is recommended. Prognosis depends on several factors, including disease stage, tumour size, depth of myometrial invasion, and molecular classifiers (ProMisE). Despite optimal treatment, the overall survival for carcinosarcoma remains poor, with most recurrences within 1 year following treatment [5]. Therefore, close surveillance is essential to facilitate early detection of recurrence and timely intervention [15].

## CONCLUSIONS

Endometrial carcinosarcoma is now regarded, staged and treated as a primary endometrial carcinoma. Early diagnosis and a multimodal treatment approach, including surgery, chemotherapy, and radiotherapy, can lead to favourable outcomes. Patients need close postoperative follow-up to detect recurrence and enable timely treatment. Application of molecular classification in all cases of endometrial carcinoma is encouraged for prognostic risk-group stratification and as a potential influence on adjuvant or systemic treatment decisions. MMRd-p53abn may have a better survival rate when a multimodal, individualised, tailored approach is followed. Novel molecular-targeted therapies could potentially improve patient care and outcomes.

## References

[1] Cantrell LA, Blank SV, Duska LR. Uterine carcinosarcoma: A review of the literature. Gynecol Oncol. 2015; 137:581–88. 10.1016/j.ygyno.2015.03.041. 25805398

[2] Toboni MD, Crane EK, Brown J, Shushkevich A, Chiang S, Slomovitz BM, Levine DA, Dowdy SC, Klopp A, Powell MA, Thaker PH. Uterine carcinosarcomas: From pathology to practice. Gynecol Oncol. 2021; 162:235–41. 10.1016/j.ygyno.2021.05.003. 34030871

[3] Berek JS, Matias-Guiu X, Creutzberg C, Fotopoulou C, Gaffney D, Kehoe S, Lindemann K, Mutch D, Concin N, and Endometrial Cancer Staging Subcommittee, and FIGO Women’s Cancer Committee. FIGO staging of endometrial cancer: 2023. Int J Gynecol Obstet. 2023; 162:383–94. https://onlinelibrary.wiley.com/doi/abs/10.1002/ijgo.14923. 37337978

[4] León-Castillo A, Gilvazquez E, Nout R, Smit VT, McAlpine JN, McConechy M, Kommoss S, Brucker SY, Carlson JW, Epstein E, Rau TT, Soslow RA, Ganesan R, et al. Clinicopathological and molecular characterisation of ‘multiple-classifier’ endometrial carcinomas. J Pathol. 2020; 250:312–22. 10.1002/path.5373. 31829447 PMC7065184

[5] Bogani G, Ray-Coquard I, Concin N, Ngoi NYL, Morice P, Caruso G, Enomoto T, Takehara K, Denys H, Lorusso D, Coleman R, Vaughan MM, Takano M, et al. Endometrial carcinosarcoma. Int J Gynecol Cancer. 2023; 33:147–74. 10.1136/ijgc-2022-004073. 36585027

[6] Erickson BK, Doo DW, Zhang B, Huh WK, Leath CA 3rd. Black race independently predicts worse survival in uterine carcinosarcoma. Gynecol Oncol. 2014; 133:238–41. 10.1016/j.ygyno.2014.02.041. 24613675

[7] Gadducci A, Cosio S, Romanini A, Genazzani AR. The management of patients with uterine sarcoma: a debated clinical challenge. Crit Rev Oncol Hematol. 2008; 65:129–42. 10.1016/j.critrevonc.2007.06.011. 17706430

[8] Li L, Huang W, Xue K, Feng L, Han Y, Wang R, Gao J. Clinical and imaging features of carcinosarcoma of the uterus and cervix. Insights Imaging. 2021; 12:142. 10.1186/s13244-021-01084-5. 34674042 PMC8531181

[9] Ravishankar P, Smith DA, Avril S, Kikano E, Ramaiya NH. Uterine carcinosarcoma: a primer for radiologists. Abdom Radiol (NY). 2019; 44:2874–85. 10.1007/s00261-019-02038-8. 31030248

[10] Huang GS, Chiu LG, Gebb JS, Gunter MJ, Sukumvanich P, Goldberg GL, Einstein MH. Serum CA125 predicts extrauterine disease and survival in uterine carcinosarcoma. Gynecol Oncol. 2007; 107:513–17. 10.1016/j.ygyno.2007.08.060. 17935762 PMC2696225

[11] Concin N, Matias-Guiu X, Vergote I, Cibula D, Mirza MR, Marnitz S, Ledermann J, Bosse T, Chargari C, Fagotti A, Fotopoulou C, Gonzalez Martin A, Lax S, et al. ESGO/ESTRO/ESP guidelines for the management of patients with endometrial carcinoma. Int J Gynecol Cancer. 2021; 31:12–39. 10.1136/ijgc-2020-002230. 33397713

[12] Travaglino A, Raffone A, Raimondo D, Arciuolo D, Angelico G, Valente M, Scaglione G, D’alessandris N, Casadio P, Inzani F, Mollo A, Santoro A, Seracchioli R, Franco Zannoni G. Prognostic value of the TCGA molecular classification in uterine carcinosarcoma. Int J Gynaecol Obstet. 2022; 158:13–20. 10.1002/ijgo.13937. 34536971 PMC9292561

[13] Powell MA, Filiaci VL, Rose PG, Mannel RS, Hanjani P, Degeest K, Miller BE, Susumu N, Ueland FR. Phase II evaluation of paclitaxel and carboplatin in the treatment of carcinosarcoma of the uterus: a Gynecologic Oncology Group study. J Clin Oncol. 2010; 28:2727–31. 10.1200/JCO.2009.26.8326. 20421537 PMC2881851

[14] Lin H, Ou YC, Fu HC, Huang SW, Wu CH, Changchien CC. Molecular subtyping and the 2023 FIGO staging in endometrial cancer: Redefining adjuvant therapy. Taiwan J Obstet Gynecol. 2025; 64:616–24. 10.1016/j.tjog.2025.04.007. 40602956

[15] Koh WJ, Abu-Rustum NR, Bean S, Bradley K, Campos SM, Cho KR, Chon HS, Chu C, Cohn D, Crispens MA, Damast S, Dorigo O, Eifel PJ, et al. Uterine Neoplasms, Version 1.2018, NCCN Clinical Practice Guidelines in Oncology. J Natl Compr Canc Netw. 2018; 16:170–99. 10.6004/jnccn.2018.0006. 29439178

